# A single cyclin–CDK complex is sufficient for both mitotic and meiotic progression in fission yeast

**DOI:** 10.1038/ncomms7871

**Published:** 2015-04-20

**Authors:** Pilar Gutiérrez-Escribano, Paul Nurse

**Affiliations:** 1 Cell Cycle Laboratory Cancer Research UK London Research Institute, London WC2A 3LY, UK; 2 Laboratory of Yeast Genetics and Cell Biology, Rockefeller University, New York, New York 10065, USA; 3 The Francis Crick Institute, London NW1 2BE, UK

## Abstract

The dominant model for eukaryotic cell cycle control proposes that cell cycle progression is driven by a succession of CDK complexes with different substrate specificities. However, in fission yeast it has been shown that a single CDK complex generated by the fusion of the Cdc13 cyclin with the CDK protein Cdc2 can drive the mitotic cell cycle. Meiosis is a modified cell cycle programme in which a single S-phase is followed by two consecutive rounds of chromosome segregation. Here we systematically analyse the requirements of the different fission yeast cyclins for meiotic cell cycle progression. We also show that a single Cdc13–Cdc2 complex, in the absence of the other cyclins, can drive the meiotic cell cycle. We propose that qualitatively different CDK complexes are not absolutely required for cell cycle progression either during mitosis or meiosis, and that a single CDK complex can drive both cell cycle programmes.

## Abstract (web summary)

It was recently shown that a single cyclin–CDK complex is sufficient to drive mitosis in fission yeast. Here, the authors show that this complex is also sufficient for meiotic progression, suggesting that specialized complexes are not required for the more intricate transitions of meiosis.

## Graphical abstract

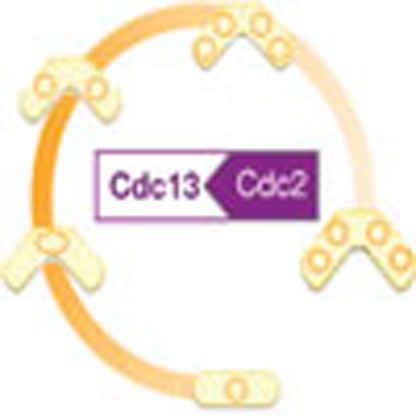

Ordered progression through the eukaryotic cell cycle is driven by cyclin-dependent protein kinase (CDK) complexes made up of a CDK catalytic subunit and an activating cyclin subunit. The dominant model for eukaryotic cell cycle control is that there is a succession of different CDK complexes with different substrate specificities that appear at different stages of the mitotic cell cycle. These qualitatively different kinase complexes drive cells through G1, S-phase, G2 and mitosis and additionally ensure there is a single S-phase each cell cycle^1^,^2^,^3^. This model has been challenged by work in fission yeast, where it has been shown that the four mitotic cell cycle CDK–cyclin complexes can be substituted by a single CDK–cyclin chimeric protein generated by the fusion of the Cdc13 cyclin with the CDK protein Cdc2 (ref. 4). These experiments have established that quantitative differences in the activity of a single CDK complex activity can bring about the different events of the cell cycle programme, and that qualitatively different CDK complexes are not absolutely required for ordered progression through the mitotic cell cycle^5^,^6^.

Meiosis is a specialized cell cycle programme in which a diploid parental cell generates haploid gametes by a single S-phase followed by two consecutive rounds of chromosome segregation. Meiotic cell cycle progression has specific features such as high levels of recombination and ploidy reduction, and involves different CDK–cyclin complexes, some of them common to the mitotic cell cycle and others that are meiosis specific. The roles of these complexes, including their functions, specificity and redundancies during the meiotic cell cycle, have not been systematically analysed.

The best-studied systems of meiotic cell cycle control are the two yeasts *Saccharomyces cerevisiae* and *Schizosaccharomyces pombe*. In the budding yeast *S. cerevisiae*, meiotic induction requires a temporally regulated gene expression cascade, which together with different post-translational regulatory mechanisms contributes to the control of cyclin protein dynamics during the meiotic cell cycle^7^,^8^. The G1 cyclins, Cln1, 2 and 3, which control the G1/S transition during the mitotic cell cycle, have no roles in the progression into premeiotic S-phase, which is instead coordinated by the meiosis-specific kinase Ime2^9^. The B-type cyclins Clb5 and Clb6, which are dispensable for the mitotic cell cycle, become essential for premeiotic S-phase^10^,^11^. The major mitotic cyclin, Clb2, is not expressed in meiosis and has no roles in the regulation of meiotic nuclear divisions^12^. Instead, meiosis I is driven by Cdc28-Clb1, Clb4 and Clb5 complexes, and meiosis II relies on the regulation of Cdc28-Clb3 and Clb5 complexes^13^,^14^,^15^. These studies suggest that the differences between the meiotic and mitotic cell cycle programmes are achieved by qualitatively different properties of various CDK complexes brought about by the different specificities conferred by the cyclin subunits.

In the fission yeast *S. pombe* the meiotic cell cycle programme is activated by nutrient deprivation and it is regulated by the Pat1 kinase^16^. Premeiotic S-phase requires the presence of at least one of the two S-phase cyclins, Cig2 and Rem1^17^,^18^, the latter being specifically expressed on meiotic induction^18^. A second meiotic-specific cyclin, Crs1, has been identified in *S. pombe* but its roles remain unclear. Crs1 was defined as a cyclin based on homology sequence analysis and no genetic or physical interaction with CDKs or other cyclins has been reported^19^,^20^. The regulation of the anaphase-promoting complex/cyclosome (APC/C), which controls Cdc13 stability, explains how CDK activity can be differentially modulated during meiotic nuclear divisions. At the end of anaphase I, the Mes1 protein antagonizes APC/C activity and prevents the degradation of a fraction of Cdc13 cyclin, which ensures there is sufficient CDK activity to progress into meiosis II^21^,^22^,^23^.

Here, we have systematically analysed the functions of the different mitotic and meiotic cyclins during the fission yeast meiotic cell cycle, identifying unreported roles for Rem1 and Crs1. In addition, using a chimeric protein approach, we demonstrate that, in the absence of other cyclins, a single CDK complex can drive ordered progression through the mitotic^4^ and meiotic cell cycle programmes.

## Results

### Requirement of Cdc13 cyclin during the meiotic cell cycle

In fission yeast, premeiotic S-phase, reductional meiosis I nuclear division and meiosis II require both mitotic- and meiotic-specific CDK–cyclin complexes^17^,^18^,^19^.

**Figure 1:**
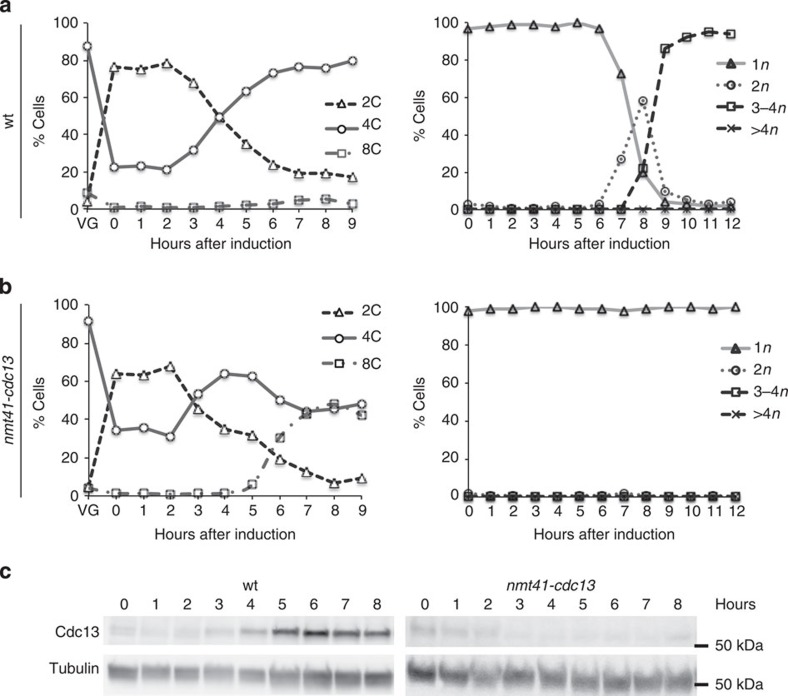
Repression of *cdc13* expression does not alter premeiotic S-phase but blocks meiotic nuclear divisions. (**a**) *pat1as2* (wt, Pi263) and (**b**) *Δcdc13 nmt41-cdc13 pat1as2 (nmt41-cdc13*, Pi313) were diploidized to get homozygous diploid strains. *cdc13* expression was repressed during nitrogen starvation by addition of thiamine and synchronous meiosis was induced. FACS samples (left panels) were taken every hour (VG: time-point before nitrogen starvation) and nuclear divisions (right panels) were followed by DAPI staining, counting the number of nuclei (*n*) of a total of 400 cells per time point. (**c**) Protein samples were analysed by western blot using anti-Cdc13 antibodies (SP4) to check the repression of *cdc13* expression and anti-tubulin antibodies (TAT1) as a loading control.

Cdc13 cyclin has been previously reported as essential for both meiosis I and II^24^,^25^, although no results about its role in premeiotic S-phase have been described. These studies used a *cdc13* temperature-sensitive mutant, *cdc13-117*, and thus experiments needed to be carried out at the restrictive temperature of 37 °C, which impairs meiotic progression in wild-type controls. Temperature-sensitive mutants can also generate variable phenotypes^26^,^27^. To avoid these limitations, and given that *cdc13* is an essential gene, we investigated the requirements of Cdc13 for meiotic cell cycle progression by switching off *cdc13* gene expression. We constructed a strain with *cdc13* under the control of the thiamine-repressible *nmt41* promoter^5^ and an ATP analogue-sensitive *pat1* allele (*pat1as2*) to induce synchronous azygotic meiosis at physiological temperatures^28^. *h+/h+ nmt41-cdc13 pat1as2* homozygous diploid strains were arrested in G1 by nitrogen starvation, and thiamine was added to repress *cdc13* expression before meiotic induction. The ATP analogue Nm-PP1 was added to the media, and samples were collected every hour and analysed by FACS to determine DNA content, and by DAPI staining to monitor nuclear divisions. An *h+/h+ cdc13+ pat1as2* grown in the same conditions was used as a control (Fig. 1a). The addition of thiamine efficiently triggered repression of the *nmt41* promoter, and *cdc13* expression was reduced when compared with the wild-type control (Fig. 1c). The decrease in Cdc13 levels did not alter the onset of premeiotic S-phase but drastically inhibited the meiotic nuclear divisions (Fig. 1b). In addition, an 8C peak was detectable in the *nmt41-cdc13* strain under repression conditions 7 h after meiotic induction, indicating that DNA re-replication was taking place after S-phase, similar to that reported for the mitotic cell cycle after *cdc13* switch off^5^. These effects were not observed in the same strain if meiosis was induced in the absence of thiamine, when only mild effects on meiosis II were observed (see Supplementary Fig. 1).

These results confirm that Cdc13 is required for meiosis I and II, and is not required for premeiotic S-phase unless this function can be achieved by very low Cdc13 protein levels.

### G1/S mitotic cyclin requirement for the meiotic cell cycle

**Figure 2:**
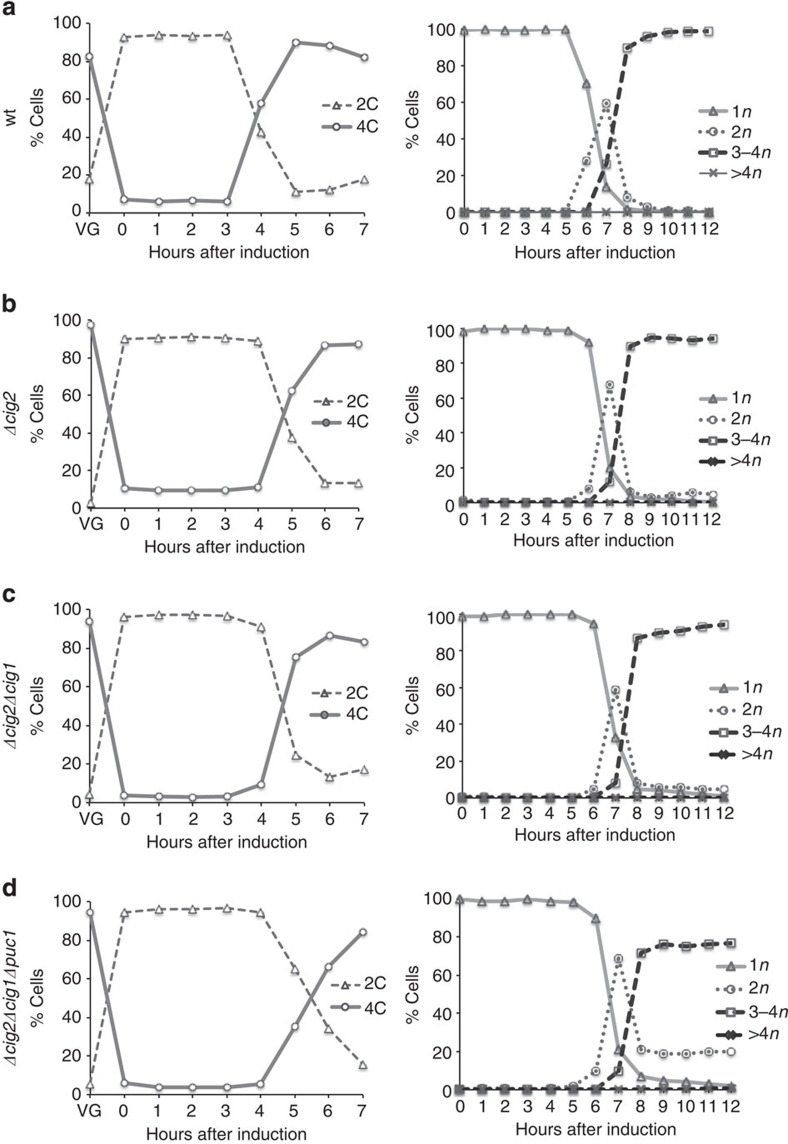
Requirement of the G1/S mitotic cyclins for the meiotic cell cycle progression. (**a**) Wild type (wt, Pi263), (**b**) Δ*cig2* (Pi336), (**c**) Δ*cig2*Δ*cig1* (Pi335) and (**d**) Δ*cig2*Δ*cig1*Δ*puc1* (Pi259), strains carrying the analogue sensitive *pat1as2* allele were diploidized and meiosis was induced after nitrogen starvation (time 0). FACS samples (left panels) were taken every hour (VG: time point before nitrogen starvation) and nuclear divisions (right panels) were followed by DAPI staining, counting the number of nuclei (*n*) of a total of 400 cells per time point.

**Figure 3:**
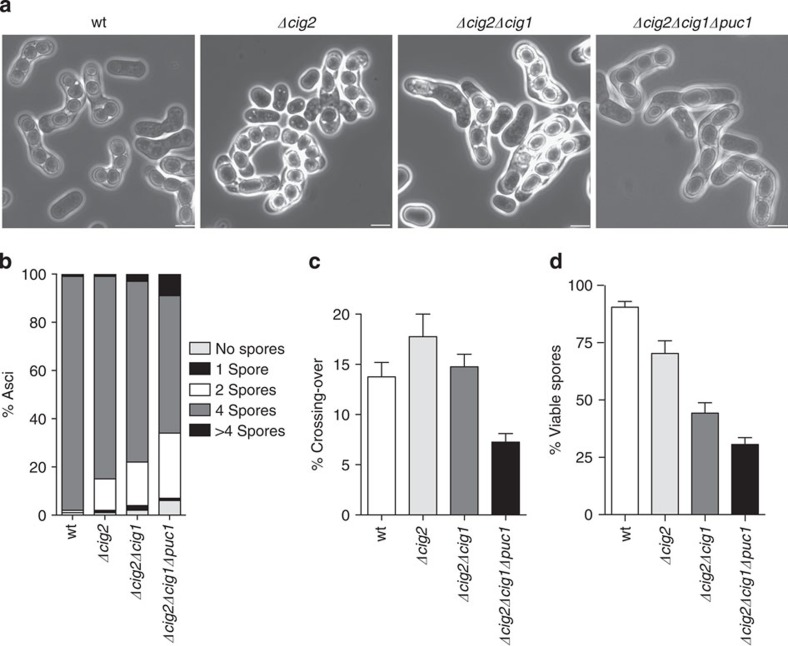
Zygotic meiosis in G1/S mitotic cyclin mutants. Zygotic meiosis was induced crossing *h+* and *h−* strains in wild type (wt, PN4 and PN71), Δ*cig2* (PN1926 and PN1931), Δ*cig2*Δ*cig1* (PN1394 and PN1401) and Δ*cig1*Δ*cig2*Δ*puc1* (Pi5 and Pi334). (**a**) Phase contrast microscopy images of each of the crosses after 48 h of incubation on MEA4S. Scale bar, 5μm. (**b**) Spore number quantification. Data correspond to a total of 400 asci from four independent crosses. (**c**) Crossing-over frequencies of the *mat1-leu1* interval. Mean values and s.e.m. from at least 400 spores from two independent crosses are indicated for each background. (**d**) Spore viability. Wild type (PN1 and PN4), Δ*cig2* (PN1926 and PN1942), Δ*cig2*Δ*cig1* (PN1400 and PN1401) and Δ*cig1*Δ*cig2*Δ*puc1* (Pi1 and Pi5) *h+* and *h−* prototroph strains were crossed, and spore viability was determined by random spore analysis (RSA) using 1,000 spores from four independent crosses. Mean values and s.e.m. are shown.

Next, we investigated the roles of the G1/S mitotic cyclins *cig1*, *cig2* and *puc1* in the meiotic cell cycle. Of these three mitotic cyclins, Cig2 is the only one for which a major role in the meiotic cell cycle has been reported; deletion of *cig2* delays progression into premeiotic S-phase and meiotic nuclear divisions, as well as causing a slight defect in meiosis II during zygotic meiosis^17^. A double *Δcig2Δcig1* mutant behaves similarly to a *Δcig2* single mutant^17^, and the Puc1 cyclin has been shown to have a role during sexual differentiation^29^ but no significant roles have been identified in meiotic cell cycle progression. Strains carrying the *cig2*, *cig1*, *puc1* triple deletion are able to mate and undergo meiosis to form spores^30^. To study the effects of these three cyclins on the timing and efficiency of progression into premeiotic S-phase and meiotic nuclear divisions, we constructed an *h+/h+ Δcig2Δcig1Δpuc1 pat1as2* homozygous diploid strain to induce synchronous azygotic meiosis. *h+/h+* wild type, *Δcig2* and *Δcig2Δcig1 pat1as2* homozygous diploid strains were used as controls. All the strains arrested efficiently in G1 and proceeded into DNA replication after Pat1 inhibition. However, the *Δcig2Δcig1Δpuc1* triple mutant showed a delay in progression into premeiotic S-phase when compared with wild type. This delay was greater than that exhibited by the *Δcig2* and *Δcig2Δcig1* strains (Fig. 2, left panels). The number of binucleated cells, corresponding to meiosis I, peaked with a similar timing in the four strains, as did meiosis II (Fig. 2, right panels). In the *Δcig2Δcig1Δpuc1* mutant, however, 20% of cells did not undergo a second nuclear division. This defect in meiosis II progression was also detected in zygotic asci generated by crosses between *h+* and *h− Δcig2Δcig1Δpuc1* haploid strains, which resulted in 27% dyads (Fig. 3a,b). This percentage was higher than that in *Δcig2* and *Δcig2Δcig1* crosses, which showed 13 and 18% dyads, respectively.

We also quantified crossing over frequencies and spore viability in zygotic meiosis (Fig. 3c,d). *Δcig2* and *Δcig2Δcig1* mutants showed levels of meiotic intergenic recombination similar to wild type. However, crossing over frequencies were significantly reduced in the *Δcig2Δcig1Δpuc1* strain. In addition, spore viability was increasingly impaired when *cig2, cig1* and *puc1* deletions were combined, from 70.3% in the *Δcig2* single mutant to 44.3% in the *Δcig2 Δcig1* mutant and 30.6% in the *Δcig2Δcig1Δpuc1* mutant.

From these results, we conclude that Cig2, Cig1 and Puc1 are additively required for normal levels of intergenic recombination and spore viability. In addition, the G1/S mitotic cyclins show minor additive roles for the onset of premeiotic S-phase and meiosis II, but overall are dispensable for efficient progression through the meiotic cell cycle progression.

### Requirement of the meiotic cyclins in the meiotic cell cycle

**Figure 4:**
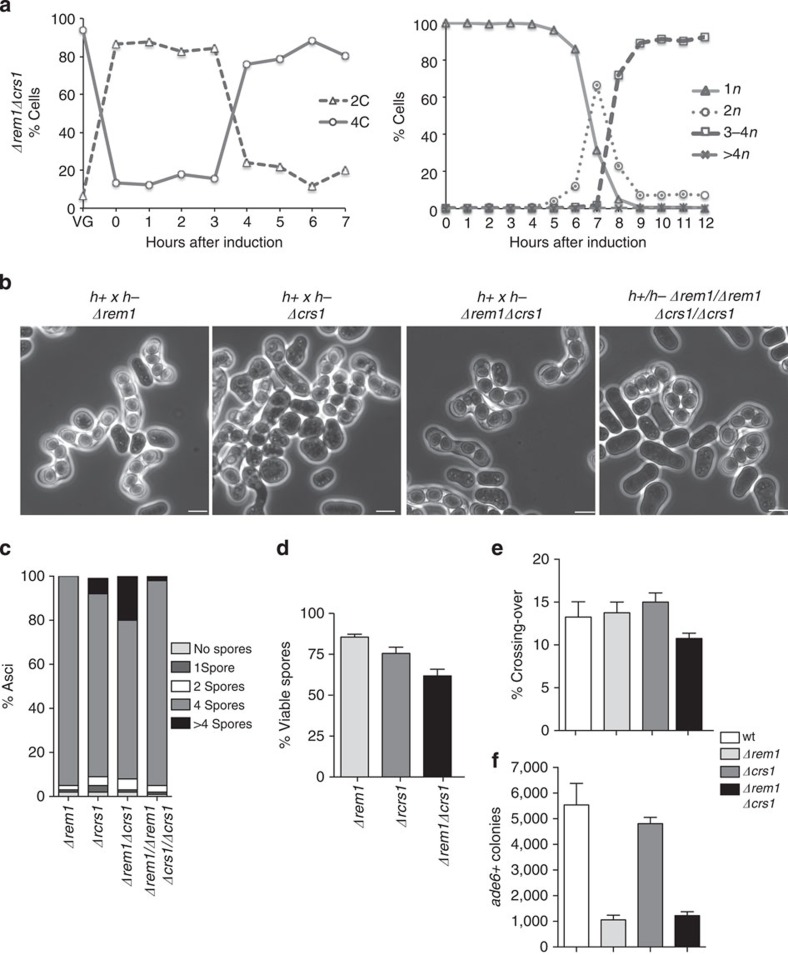
Requirement of *rem1* and *crs1* meiosis specific cyclins for the meiotic cell cycle programme. (**a**) Synchronous azygotic meiosis in an h+/h+ *Δrem1Δcrs1 pat1as2* homozygous diploid strain. Cell samples were taken every hour to follow premeiotic DNA replication by FACS (left panel) and nuclear content by DAPI staining (right panel). (**b**) Phase contrast microscopy images of zygotic asci from *Δrem1* (Pi163 and Pi337), *Δcrs1* (Pi166 and Pi339), *Δrem1Δcrs1* (Pi175 and Pi176) crosses and azygotic asci from an h+/h− *Δrem1Δcrs1* homozygous diploid (Pi230). Scale bar, 5μm. (**c**) Spore number per ascus. Data correspond to a total of 400 asci from four independent crosses. (**d**) Spore viability of spores from zygotic meiosis. Colony formation was quantified after RSA using 1,000 spores from four independent crosses. Mean percentage values and s.e.m. are shown. (**e**) Crossing-over frequencies of the *mat1-leu1* interval. Mean values and s.e.m. from at least 400 viable spores per genetic background are represented. (**f**) Intragenic *ade6* recombination (*ade6-M210ade6M216*) of wild type (wt, PN71 and Pi333), *Δrem1* (Pi340 and Pi344), *Δcrs1* (Pi327 and Pi328) and *Δrem1Δcrs1* (Pi346 and Pi348). Total numbers of Ade6^+^ colonies per 10^6^ spores are shown.

We next considered the functions of the meiosis specific cyclins on meiotic cell cycle progression using an *h+/h+ Δrem1Δcrs1 pat1as2* homozygous diploid strain. As shown in Fig. 4a, the 4C peak corresponding to premeiotic S-phase appeared with a similar timing to that observed for wild type (Fig. 2a). Progression into meiosis I and II also followed an identical time course to wild type (Figs 4a and 2a, right panels), showing that the Rem1 and Crs1 cyclins have no essential roles in meiotic cell cycle progression when the mitotic cyclins are present.

We did not detect any defects in progression into meiotic nuclear divisions during synchronous azygotic meiosis, but previous studies have reported that a *Δrem1Δcrs1* double mutant shows an increase in diploid progeny on zygotic meiosis induction^19^. Zygote formation requires the conjugation of the haploid parental cells before proceeding into the meiotic cell cycle, and so this additional stage could explain these phenotypic differences. We constructed *h+* and *h- Δrem1Δcrs1* haploid strains and crossed them in sporulation media to induce zygotic meiosis. The majority of the asci (72%) contained four spores, while the remaining 28% showed an abnormal spore number, mainly more than four spores (20%). *Δrem1* and *Δcrs1* single mutants were used as controls and showed no major defects in the number of spores per ascus. This phenotype was also not detected when azygotic meiosis was induced using an *h+/h− Δrem1Δcrs1* diploid strain (Fig. 4b,c), indicating that the role of Rem1 and Crs1 in the regulation of the spore number is specific for zygotic meiosis. As previously described^19^, *Δrem1Δcrs1* crosses exhibited reduced spore viability when compared with the wild type and the single-deletion controls (Fig. 4d). To further investigate the defects in spore number, we carried out live-cell imaging of *h*^*90*^ *Δrem1Δcrs1* strains in which chromatin was visualized using Hht1-CFP, and microtubules and spindle pole body (SPB) were visualized using Atb2-mCherry and Sid4-mCherry, respectively. On meiotic induction, no defects in karyogamy were detected, but 19.6% of the analysed zygotes (*n*=112) exhibited abnormal meiotic nuclear divisions that resulted in three nuclear masses instead of two. In 86.4% of the aberrant divisions, the Sid4 signal monitoring the SPBs was split into three dots, instead of two (Supplementary Fig. 2, left panel, 120 min and right panel, 40 min, respectively), suggesting the presence of multipolar SPBs. We conclude that the Rem1 and Crs1 cyclins are involved in the regulation of meiotic nuclear divisions but only during zygotic meiosis.

Next, we quantified the efficiency of both inter and intragenic recombination in *rem1* and *crs1* single and double deletion backgrounds. We did not detect major changes in the crossing over frequencies in any of the strains analysed. Only the *Δrem1Δcrs1* double mutant showed a minor decrease in its intergenic recombination percentage (Fig. 4e). These results suggest that the meiosis-specific cyclins have no essential roles in the regulation of intergenic recombination. *ade6* gene conversion showed similar levels in wild type and *Δcrs1* strains while, as reported before^18^, it was significantly reduced in the *Δrem1* mutant (Fig. 4f). Similar defects were observed in *Δrem1Δcrs1* crosses, indicating that Rem1 and Crs1 do not have additive roles in the regulation of intragenic recombination.

We conclude that, despite having roles in the regulation of meiotic recombination and nuclear division during zygotic meiosis, Rem1 and Crs1 do not have major roles in azygotic meiosis and so are not strictly required for meiotic cell cycle progression.

### Requirement of mitotic and meiotic cyclins during meiosis

**Figure 5:**
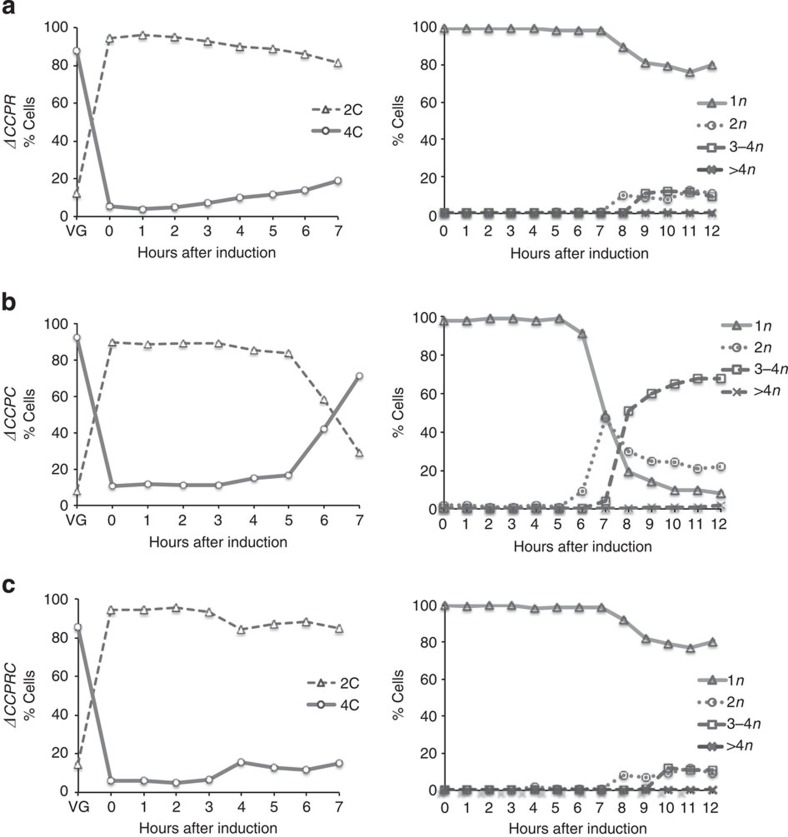
Mitotic- and meiotic-specific cyclins are additively required for meiotic cell cycle progression. (**a**) Δ*cig2*Δ*cig1*Δ*puc1*Δ*rem1* (*ΔCCPR*, Pi240), (**b**) Δ*cig2*Δ*cig1*Δ*puc1*Δ*crs1* (*ΔCCPC*, Pi296) and (**c**) Δ*cig2*Δ*cig1*Δ*puc1*Δ*rem1*Δ*crs1* (*ΔCCPRC*, Pi278), strains were treated as in Figs 2 and 4a to induce synchronous meiosis. DNA (left panels) and nuclei number per cell (right panels) were followed every hour after meiosis induction.

Next, we analysed the effect of *rem1* and *crs1* deletions in a *Δcig2Δcig1Δpuc1* background during meiotic cell cycle progression to study the redundancies between both groups of cyclins. It has been reported that *Δcig2Δrem1* double mutants are blocked in meiotic cell cycle progression before DNA replication, although the meiosis-specific transcription programme is activated^18^. Simultaneous deletion of *cig2*, *cig1*, *puc1* and *rem1 (ΔCCPR),* or *cig2*, *cig1*, *puc1*, *rem1* and *crs1 (ΔCCPRC)* in *h+/h+ pat1as2* homozygous diploid strains, blocked progression into premeiotic S-phase. In both strains, <20% of the population subsequently underwent DNA replication and nuclear division (Fig. 5a,c).

We also analysed the effect of deleting *crs1* in the absence of the G1/S mitotic cyclins. In contrast to the *ΔCCPR* and *ΔCCPRC* mutant strains, simultaneous deletion of *cig2*, *cig1*, *puc1* and *crs1 (ΔCCPC)* did not block progression into premeiotic S-phase; more than 70% of the *ΔCCPC* population underwent DNA replication (Fig. 5b). However, this strain did show a delay in the onset of premeiotic S-phase as well as defects in meiosis II progression resulting in 20% dyads formation (Fig. 5b), a phenotype similar to the *Δcig2Δcig1Δpuc1* triple mutant (Fig. 2d). In addition, in an *h+/h+ ΔCCPC pat1as2* homozygous diploid strain the percentage of cells with only one nucleus after 12 h of meiotic induction was higher (12%) than that in the *Δcig2Δcig1Δpuc1* background (1%), suggesting that *cig2*, *cig1*, *puc1* and *crs1* have additive roles in initial steps of meiotic cell cycle progression.

**Figure 6:**
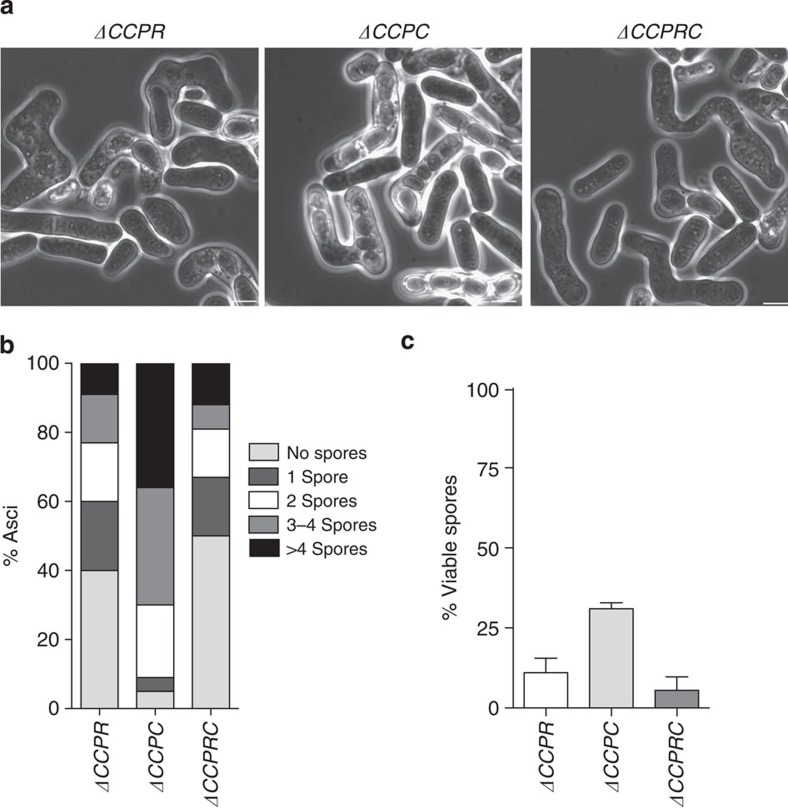
Zygotic meiosis in mitotic and meiotic cyclin deletion mutants. (**a**) Phase contrast microscopy images of zygotic asci from Δ*cig2*Δ*cig1*Δ*puc1*Δ*rem1* (*ΔCCPR*, Pi236 and Pi251), Δ*cig2*Δ*cig1*Δ*puc1*Δ*crs1* (*ΔCCPC*, Pi237 and Pi292) and Δ*cig2*Δ*cig1*Δ*puc1*Δ*rem1*Δ*crs1* (*ΔCCPRC*, Pi211 and Pi242). Scale bar, 5μm. (**b**) Spore number per ascus (*n*=400 asci per each genetic background). (**c**) Spore viability (*n*=1,000 spores per each genetic background).

Given the roles of Rem1 and Crs1 cyclins in nuclear division during zygotic meiosis, we analysed spore number per ascus in *ΔCCPR, ΔCCPC* and *ΔCCPRC* zygotic asci. In the case of the *ΔCCPR* and *ΔCCPRC* strains, the majority of the asci (60% for *ΔCCPR* and 67.4% for *ΔCCPR*C) were either empty or contained only one spore, similar to the defects in meiotic cell cycle progression described for these strains during azygotic meiosis. It was also found that 17.1% of *ΔCCPR* and 13.9% of *ΔCCPRC* zygotic asci contained two spores, 14.3 and 7%, 3–4 spores, and 8.6 and 11.6%, more than four spores per ascus (Fig. 6a,b). For both crosses, spore viability was significantly reduced (11% for *ΔCCPR* and 5.5% for *ΔCCPRC*). In the *ΔCCPC* crosses, spore viability was 31% (Fig. 6c), similar to the 32% described for the *Δcig2Δcig1Δpuc1* crosses. In addition, 20% of the asci were dyads, similar to that described for azygotic meiosis (Fig. 5b). In all, 10% of the asci were either empty or had one spore, while 36% of the asci contained more than four spores. This phenotype was not detected in the *Δcig2Δcig1Δpuc1* or *Δ*crs1 mutant strains, indicating that both mitotic- and meiosis-specific cyclins have additive effects in the regulation of nuclear division during zygotic meiosis.

We conclude that in fission yeast mitotic and meiotic cyclins are redundantly required for meiotic cell cycle progression. Our results also indicate a previously unappreciated role of mitotic- and meiosis-specific cyclins in zygotic meiosis.

### Meiotic cell cycle progression based on a single CDK–cyclin

Proper progression through the meiotic cell cycle requires a range of different mitotic and meiotic cyclins. However, although there are four different CDK–Cyclin complexes operative in the mitotic cell cycle, it has been shown that a single CDK–cyclin can drive the mitotic cell cycle programme. Different thresholds of CDK activity promote the orderly progression of the different events required for DNA replication and cell division^4^,^5^. Therefore, we investigated whether we could engineer a single CDK–cyclin complex that could also drive the meiotic cell cycle programme. Our starting point was a fusion protein between Cdc13 and Cdc2 (ref. 4). The characteristics of this fusion protein are that the cyclin and kinase subunits are present in a 1:1 ratio and their interaction is not altered by differential spatial regulation of the subunits; the fused expression of the two proteins removes the need for functions bringing about complex formation and the fusion protein is likely to prevent the association of Cdc2 with other cyclins.

**Figure 7:**
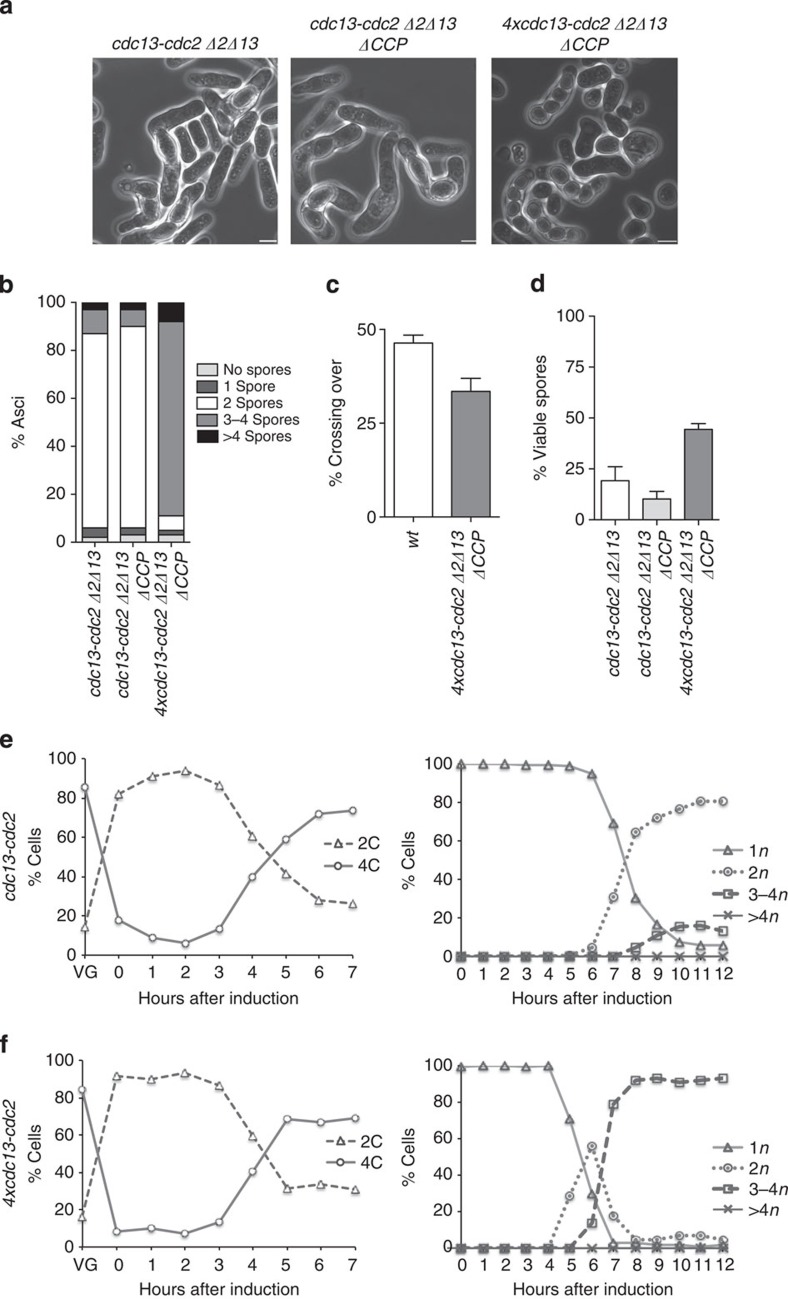
Meiotic cell cycle programme driven by a Cdc13–Cdc2 fusion protein. Zygotic meiosis was induced crossing *h+* and *h−* strains in *cdc13-cdc2 Δcdc2Δcdc13* (DC210 and DC217), *cdc13-cdc2 Δcdc2Δcdc13ΔCCP (*Pi4 and DC235), *4xcdc13-cdc2 Δcdc2Δcdc13ΔCCP* (Pi43 and Pi46). (**a**) Phase contrast microscopy images of the asci after 48 h of incubation on MEA4S. Scale bar, 5 μm. (**b**) Spore number per ascus (*n*=400 asci per strain). (**c**) Crossing-over frequencies of the *mat1-cdc13* interval (*n*=400 viable spores per strain). (**d**) Spore viability (*n*=1,000 spores per strain). (**e**) *cdc13-cdc2 Δcdc2Δcdc13ΔCCP* (Pi253), (**f**) *4xcdc13-cdc2Δcdc2Δcdc13ΔCCP* (Pi260) strains carrying the analogue-sensitive *pat1as2* allele were treated as described in Fig. 2 to study meiotic cell cycle progression. DNA content (left panel) and the number of nuclei per asci (right panel) are shown.

We tested the ability of the Cdc13-Cdc2 protein expressed under *cdc13* regulatory regions to drive meiotic cell cycle progression by crossing *h+* and *h− cdc13-cdc2* strains in which endogenous *cdc2* and *cdc13* were deleted (*Δ2Δ13*). Spore number per ascus analysis showed that the crosses expressing the Cdc13-Cdc2 chimera resulted in 85% of the asci containing two spores rather than four with a low percentage of viability of <20%. Deletion of the G1/S mitotic cyclins (*ΔCCP*) in this background resulted in the same phenotype (Fig. 7a,b and d). To characterize the phenotypic effects of the chimeric CDK protein in the meiotic cell cycle, analyses of premeiotic S-phase and nuclear division progression were carried out using an *h+/h+ cdc13-cdc2 Δ2Δ13ΔCCP pat1as2* homozygous diploid strain (Fig. 7e). G1 arrest upon nitrogen starvation was followed by DNA replication and a first round of nuclear division 4 and 7 h after induction of meiosis, respectively. However, the Cdc13-Cdc2 chimera failed to efficiently undergo a second meiotic nuclear division, which occurred in <20% of the population, consistent with the spore number per ascus. These results show that the expression of Cdc13-Cdc2 chimeric protein as the only source of CDK activity can drive premeiotic S-phase but cannot complete the meiotic nuclear divisions.

Dyad formation on meiotic induction has been reported to be the result of mutations that compromise CDK activity, such as different *cdc2* thermo-sensitive alleles^4^,^25^,^26^,^27^, or the deletion of the APC/C antagonist *mes1*, which results in premature degradation of Cdc13 at the end of meiosis I, preventing the cells undergoing a second meiotic division^21^,^22^,^23^. Given these observations, we analysed the dynamics of Cdc13-Cdc2 protein levels on induction of synchronous azygotic meiosis and compared them with the endogenous Cdc13 and Cdc2 in a wild-type strain. As described in previous studies^17^,^23^, endogenous Cdc2 levels remained constant in the control strain through the whole meiotic cycle. Cdc13 protein began to accumulate at the onset of premeiotic DNA replication (3 h) until the beginning of the second nuclear division (7 h). In the chimeric strain, *cdc13-cdc2* expression differed from that observed for endogenous *cdc13* in the wild type. Protein accumulation began 1 h later than in the wild type, although the timing of disappearance was similar for both proteins (Supplementary Fig. 3). Therefore, the inability of the Cdc13-Cdc2 protein to drive progression into the two consecutive meiotic nuclear divisions could be the result of delayed protein accumulation possibly due to the rate of synthesis being too low. To investigate this, we increased the expression levels of *cdc13-cdc2* chimera to determine whether this would correct the meiosis II arrest. Four copies of the *cdc13-cdc2* fusion under endogenous *cdc13* regulatory elements were integrated in a *Δ2Δ13ΔCCP* background. The increase in copy number resulted in an increase in the protein levels (Supplementary Fig. 4a). This probably led to an increase in the level of CDK activity as indicated by the reduction of cell size at division of these strains during the mitotic cell cycle when compared with single *cdc13-cdc2* strains (Supplementary Fig. 4b). FACS profiles and generation times of these strains were unaltered when compared with the wild type and *cdc13-cdc2* parental strains (Supplementary Fig. 4c,d).

Crosses between *h+* and *h−* 4*xcdc13-cdc2 Δ2Δ13ΔCCP* strains resulted in more than 80% four-spored asci (Fig. 7b). Crossing-over frequency was analysed at the *mat1-cdc13* interval and showed a modest reduction to 33% when compared with the 46% of the wild type (Fig. 7c). Overexpression of the Cdc13-Cdc2 chimera significantly increased spore viability from the 9% described for the 1*xcdc13-cdc2 Δ2Δ13ΔCCP* crosses to 46% (Fig. 7d). In azygotic meiosis, expression of four copies of *cdc13-cdc2* fusion in an *h+/h+ Δ2Δ13ΔCCP pat1as2* homozygous diploid background restored meiotic progression. Premeiotic S-phase and meiosis I and II had similar timing to the control strain after Pat1 inactivation, although there was a slight advance of both meiotic nuclear divisions (Fig. 7f). No defects in DNA segregation were identified when the DNA content of the asci was visualized by DAPI staining (Supplementary Fig. 5). Therefore, efficient progression through the main events of the meiotic cell cycle can be brought about by increasing the chimeric protein gene copy number. This is correlated with a pattern of protein accumulation which is similar to that observed for the endogenous Cdc13 protein in the wild-type control strain (Supplementary Fig. 3).

**Figure 8:**
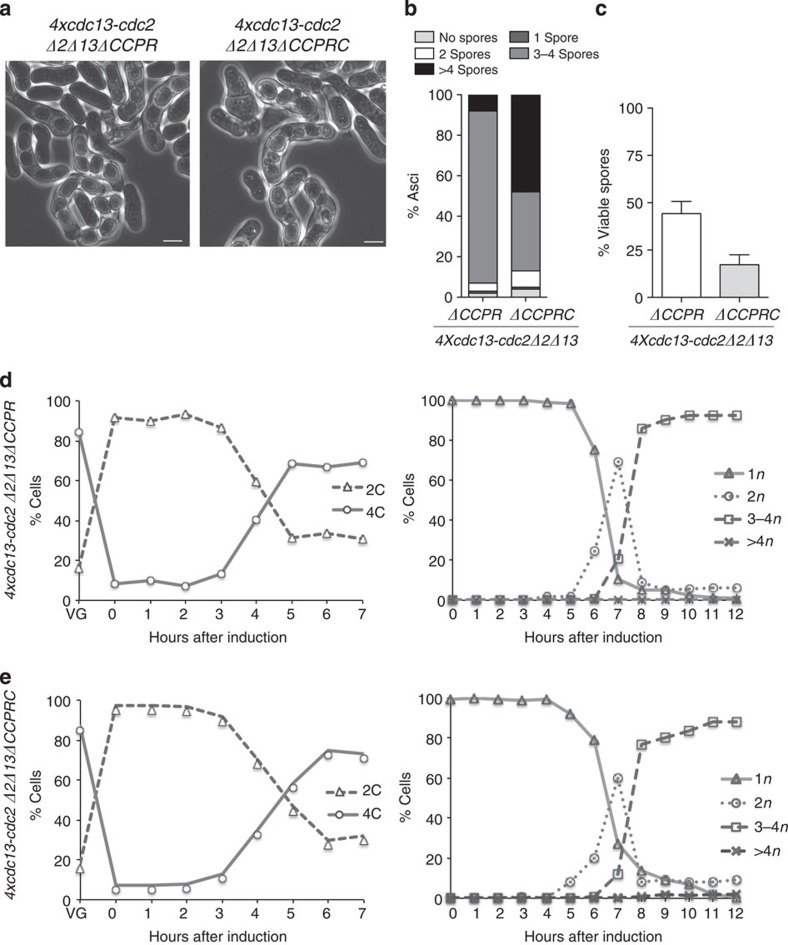
Overexpression of Cdc13-Cdc2 fusion allows meiotic progression in the absence of endogenous cyclins. (**a**) Phase contrast images of zygotic asci from 4 × *cdc13-cdc2* Δ*2*Δ*13*Δ*CCPR* (Pi130 and Pi132) and 4 × *cdc13-cdc2* Δ*2*Δ*13*Δ*CCPRC* (Pi155 and Pi158) crosses. Scale bar, 5 μm. (**b**) Spore number per ascus (*n*=400 asci per genetic background). (**c**) Spore viability (*n*=1,000 spores per genetic background). Meiotic cell cycle progression was analysed in synchronous azygotic meiotic cultures treating 4 × *cdc13-cdc2* Δ*2*Δ*13*Δ*CCPR* (Pi279), (**d**) and 4 × *cdc13-cdc2* Δ*2*Δ*13*Δ*CCPRC* (Pi285), (**e**). DNA content (left panel) and the number of nuclei per asci (right panel) are shown.

We constructed a strain in which Rem1 and Crs1 were deleted to test for any effect of the meiotic cyclins on the completion of meiotic cell cycle progression in the 4*xcdc13-cdc2* strain. Simultaneous deletion of *cig2, cig1, puc1* and *rem1* in a *cdc13+ cdc2+* background blocked progression into premeiotic S-phase (Fig. 5a). In contrast, deletion of these four cyclins (*ΔCCPR)* in a *4xcdc13-cdc2* background had no effect on entry into premeiotic S-phase or the two meiotic nuclear divisions in either zygotic or azygotic meiosis. Progression into the meiotic cell cycle was also unaltered in an *h+/h+* 4*xcdc13-cdc2 Δ2Δ13ΔCCPRC pat1as2* homozygous diploid strain during azygotic meiosis (Fig. 8). However, induction of zygotic meiosis using *h+* and *h−* 4*xcdc13-cdc2 Δ2Δ13ΔCCPRC* strains produced asci with defects in spore number per ascus, as shown in Fig. 8a,b, in which more than 60% of the population showed more than four spores per ascus with reduced spore viability (Fig. 8c) This result is consistent with the results obtained for the *Δrem1Δcrs1* double mutant in which meiotic nuclear divisions were altered during zygotic meiosis.

Our results indicate that a range of qualitatively different mitotic and meiotic CDK-cyclin complexes is not strictly required for orderly progression through premeiotic S-phase, meiosis I and meiosis II, and that appropriate expression of a single CDK-cyclin chimera is able to efficiently drive progression through both the mitotic and meiotic cell cycle programmes in the absence of other CDK activities.

## Discussion

We have systematically investigated the requirements of the six fission yeast cyclins for the meiotic cell cycle programme. Four of these cyclins, *cdc13*, *cig2*, *cig1* and *puc1*, are common to the mitotic cycle and the other two, *rem1* and *crs1*, are meiotic cell cycle specific. We confirmed that the Cdc13 cyclin is essential for both the mitotic and meiotic cell cycles^24^,^25^,^31^,^32^. We have shown that, in meiosis, repression of *cdc13* expression does not have a major effect on premeiotic S-phase progression, but does block the two meiotic nuclear divisions and induces re-replication (Fig. 1b). In this respect, the meiotic cell cycle requirements for *cdc13* are similar to those observed for the mitotic cell cycle, in which cells lacking Cdc13 can undergo S-phase but not mitosis, and activate extra rounds of DNA replication^5^,^31^.

None of the remaining five cyclins are absolutely essential for meiotic cell cycle progression. However, their deletion results in varying degrees of impairment at different stages of the meiotic cell cycle programme affecting both recombination and spore viability. These defects become more severe when different cyclin deletions are combined together: a *Δcig2Δcig1Δpuc1* triple mutant shows minor defects on progression through premeiotic S-phase and meiosis II. These defects are further enhanced if the two meiosis-specific cyclins are deleted in a *cig2Δcig1Δpuc1Δ* background. The onset of premeiotic S-phase is greatly delayed in a *cig2Δcig1Δpuc1Δcrs1Δ* mutant while both the *cig2Δcig1Δpuc1Δrem1Δ* and *cig2Δcig1Δpuc1Δrem1Δcrs1Δ* strains became completely blocked before premeiotic DNA synthesis (Fig. 5). We propose that the block over premeiotic DNA synthesis is due to *cig2* and *rem1* being deleted, as premeiotic S-phase has been shown to require at least one of these two cyclins^18^.

We also identified a novel role for the two meiotic cyclins *rem1* and *crs1* during zygotic meiosis initiated by conjugation of h+ and h− haploid cells. *rem1Δcrs1Δ* double mutants exhibited aberrant nuclear divisions that resulted in 20% of asci containing more than four spores (Fig. 4c). Mitotic cyclins could also contribute to this regulation, given that the combined deletion of the *cig2, cig1* and *puc1* with the meiosis specific cyclins, resulted in different percentages of asci with more than four spores depending on the particular combination of the cyclin deletions (Fig. 6).

Our systematic analysis has revealed the complexity of cyclin requirements for meiotic cell cycle progression in wild-type cells. Five of the cyclins studied, Cdc13, Cig2, Cig1, Puc1 and Rem1, have been shown to form active complexes with Cdc2, although Crs1 has not. Our results indicate that the different CDK–cyclin complexes act together to bring about the meiotic cell cycle programme in wild-type cells. A scenario could be imagined in which various CDK complexes function at different steps of the meiotic programme, phosphorylating different substrates at various times to ensure orderly progression through the meiotic cell cycle. When one or more of the cyclin genes are deleted, progression through the various steps of the meiotic cell cycle becomes increasingly impaired. This scenario is similar to that which is generally thought to operate during the mitotic cell cycle.

It has been shown that all the cyclins involved in the fission yeast mitotic cell cycle can be replaced by a single CDK–cyclin generated by a fusion between Cdc13 and Cdc2 (ref. 4). This suggests that it is not qualitative differences between the different CDK–cyclin complexes that drive progression through the mitotic cell cycle programme, but rather quantitative differences in CDK activity levels that are important. Given this alternative view of the mitotic cycle we tested whether a single CDK–cyclin complex could also drive the programme of the more complex meiotic cell cycle. We found that the Cdc13-Cdc2 fusion was unable to undergo the second meiotic nuclear division but did efficiently progress into premeiotic S-phase and meiosis I (Fig. 7e). However, when the Cdc13-Cdc2 fusion was expressed at a higher level, both the mitotic and the azygotic meiotic cell cycle programmes were carried out efficiently in over 80% of the cells, even though all the other cyclins were deleted (Fig. 8e).

In this work, we have shown that, although endogenous Cdc13 is dispensable for DNA replication in the meiotic cell cycle (Fig. 1b), it is not sufficient for premeiotic S-phase to take place when the other cyclins are absent (Fig. 5c). In contrast, the Cdc13–Cdc2 fusion protein construct was able to efficiently drive premeiotic S-phase in a *ΔCCPRC* background (Fig. 8e). This result indicates that, as in the mitotic cycle, Cdc13 has the ability to drive DNA replication during meiosis. We propose that the particular regulation of endogenous Cdc13 and Cdc2 during premeiotic S-phase in wild-type cells, which could involve protein localization, complex formation or the action of specific inhibitors among other possibilities, prevents the proper modulation of protein kinase activity needed to bring about the meiotic cell cycle programme in the absence of other CDK complexes. In contrast, fusion of Cdc13 and Cdc2 allows the Cdc13–Cdc2 complex to phosphorylate its substrates and drive progression through the meiotic cell cycle programme without other CDK complexes.

During zygotic meiosis, the absence of *rem1* and *crs1* in a *4xcdc13-cdc2* background triggered an increase in the number of asci with more than four spores. This increase in spore number per ascus was not detected in the Cdc13–Cdc2 fusion protein overexpression strain when *rem1* and *crs1* were still present, even though the fusion of Cdc13 and Cdc2 is likely to avoid the interaction of Cdc2 with endogenous cyclins. This suggests that the Rem1 and Crs1 functions during zygotic meiosis may not rely on their interaction with Cdc2. CDK-independent roles of cyclin subunits have been reported in higher eukaryotes, in which cyclin D1 regulates transcriptional activity independently of Cdk4 catalytic activity (reviewed in ref. 33). Use of the Cdc13–Cdc2 chimeric protein as the sole source of CDK activity will be useful to investigate cyclin functions independent of interactions with the CDK catalytic subunit.

We showed that the overexpression of Cdc13–Cdc2 fusion as the sole source of CDK activity is able to drive progression into premeiotic S-phase, meiosis I and meiosis II. However, the reduction in meiotic intergenic recombination and spore viability observed suggests that specific CDK–cyclin complexes could be required for fine regulation of specific events of the meiotic cell cycle programme.

It is possible that in the primitive founder eukaryote there was only a single CDK–cyclin complex with one CDK and one cyclin subunit. Over evolutionary time, extra cyclins and extra CDKs evolved to fine tune progression through the mitotic and meiotic cell cycles. Selective pressure could have made these cell cycle programmes more robust, dependent on more complex networks of CDK–cyclin complexes and introducing differences between the two cell cycle programmes. The cell could have then become addicted to this more complex CDK–cyclin network system such that the original CDK–cyclin complex was no longer effective at driving the mitotic and meiotic cell cycles. However, introduction of the Cdc13–Cdc2 fusion protein at an appropriate level of expression removed the dependence of the cell on multiple cyclin networks.

Our results indicate that qualitatively different CDK–cyclin complexes are not absolutely required for the basic meiotic cell cycle programme. We propose that the quantitative model for mitotic cell cycle progression^4^,^5^,^6^ also applies to the more complex meiotic cell cycle, suggesting that the main events of both cell cycle programmes can be brought about by different levels of CDK–cyclin complex activity. Given the plasticity observed in the requirements for various CDK–cyclin complexes in other eukaryotes (reviewed in refs 34, 35, 36), we propose that this quantitative model for CDK cell cycle control may also apply to eukaryotes more complex than fission yeast, including the metazoa.

## Methods

### Strains and growth conditions

The strains used in this study are listed the Supplementary Table 1. Media preparation and strain construction were done following standard methods^37^,^38^. The *Δcig1::ura4*^*+*^*, Δcig1::ura4*^*+*^*, Δpuc1::ura4*^*+*^*, Δcdc13::natMX6, Δcdc2::kanMX6* and *Δleu1::cdc13-cdc2-ura4*^*+*^ strains have been previously described^4^,^5^,^29^. *Δrem1 and Δcrs1* deletions using *natNT2* and *hphNT1* markers were replacements of the open-reading frames (ORF)^39^,^40^. Fluorescent proteins were constructed as previously reported^41^,^42^. For the overexpression of *cdc13-cdc2* chimera, integration of extra copies of the construction at the *ura4* locus was achieved by transformation of *cdc13-cdc2 ΔCCP* strains with a plasmid in which *cdc13-cdc2-ura4+* construction was cloned adjacent to *Scleu2*, linearized by StuI digestion at the *ura4* marker. Quantification of *cdc13-cdc2* copy number was done by qPCR from genomic DNA of different *leu2*^*+*^ clones. *pat1as2* strains were constructed replacing the wild-type *pat1* ORF by the mutant allele using *hph* as a selection marker^28^. Transformants were tested for Pat1 chemical inactivation on YE4S plates supplemented with 30 μM of the Nm-PP1 inhibitor (TRC) from a 10 mM stock in DMSO. Homozygous diploids were obtained inducing diploidization of haploid strains by incubating cells in YE4S with 20 μg ml^−1^ of Carbendazim (MBC, Sigma, 2 mg ml^−1^ stock in DMSO) for 5 h at 25 °C. Cells were plated on YE4S containing 5 μg ml^−1^ of Phloxin B. Dark pink colonies were selected and DNA content was analysed by flow cytometry.

### Zygotic meiosis

*h+* and *h−* strains were crossed on ME4S plates and incubated at 25 °C for 48 h. The resulting asci were visualized by phase contrast microscopy using a Zeiss Axioskop 40 microscope equipped with a 63 × /1.4 NA objective and a Zeiss AxioCam MRm camera. At least 400 asci were counted under the microscope for spore number quantification. Cell viability was calculated by plating 250 spores obtained by random spore analysis on YE4S plates in duplicate and counting the number of colonies after 48–72 h of incubation. At least two independent crosses were used for each genetic background (*n*=1,000 spores).

### Synchronous azygotic meiosis

Single colonies of diploid homozygous strains carrying the *pat1as2* allele were exponentially grown in YE4S but in the case of *nmt41-cdc13* experiment series in which the strains were grown in EMM4S at 25 °C to a final concentration of 6.3 × 10^6^ cells ml^−1^. Cultures were then filtered through a Millipore membrane, washed with 3 volumes of EMM-NH_4_Cl and resuspended in EMM-NH_4_Cl supplemented with 150 μg ml^−1^ of leucine. Cells were grown at 25 °C for 17 h, filtered again and resuspended in fresh EMM-NH_4_Cl media supplemented with leucine. For Pat1 chemical inactivation and meiosis induction, the Nm-PP1 inhibitor was added to the cultures at a final concentration of 30 μM and cells were grown at 28 °C. Repression of the *nmt41* promoter was achieved by addition of 5 μg ml^−1^ of thiamine to the media, 2 h before meiotic induction.

### Flow cytometry

DNA content analysis was performed by flow cytometry from 70% ethanol-fixed cells resuspended in 50 mM of sodium citrate and 2 μg ml^−1^ of propidium-iodine^43^ using a BD FACS Fortessa.

### DAPI staining

For each meiotic time point, DNA was visualized in heat-fixed cells using 4′,6-diamidino-2-phenylindole (DAPI, Sigma) at a final concentration of 2 μg ml^−1^. For nuclei quantification, 400 cells were counted under a Zeiss Axioskop 40 microscope equipped with a × 63/1.4 NA objective and a Zeiss AxioCam MRm camera.

### Protein extraction and western blot

Total protein extracts were prepared from 10^8^ cells fixed with 10%TCA. Fixed samples were washed with acetone and dried at −80 °C for at least 1 h. Dry pellets were resuspended in 100 μl of breaking buffer (8 M Urea, 50 mM Ammonium Bicarbonate and 5 mM EDTA), containing protease inhibitors (Complete Mini protease inhibitor cocktail) and phosphatase inhibitors (Roche PhosStop). Cell suspensions were transferred to a tube containing 750 μl of glass beads (0.4 mm; Sigma) and disrupted in a FastPrep cell disruptor (ThermoSavant) for 3 × 45 s. Breaking buffer plus inhibitors (75 μl) was added, and the crude extract was recovered and mixed with 4 × sample buffer (20% (v/v) β-mercaptoethanol, 20% (w/v) SDS, 0.05% (w/v) bromophenol blue, 25% (v/v) glycerol, 300 mM Tris-HCl pH6.8). Finally, extracts were boiled for 5 min and centrifuged at 13,000 r.p.m. for 1 min. In western blots, Cdc13 and Cdc13–Cdc2 fusion protein were probed with a rabbit polyclonal SP4 antibody^44^ (1:5,000); Cdc2 with commercial rabbit polyclonal anti-PSTAIRE (1:500; Santa Cruz Biotech); phosphorylated Tyr15 Cdc2 with commercial rabbit polyclonal (1:500; Cell Signaling Technology); and α-tubulin with monoclonal TAT1 antibody (1:5,000; gift from K Gull^45^). Horseradish peroxidase-conjugated goat anti-mouse or goat anti-rabbit IgG (Pierce-Thermo) were used at a dilution of 1:10,000 as secondary antibodies.

### Live cell imaging

For time-lapse microscopy, h^90^ strains were incubated on MEA4S plates at 29 °C for 8 h, transferred to MatTek dishes containing 3 ml of EMM-NH_4_Cl and visualized using a DeltaVision Elite (Applied Precision) comprising an Olympus IX71 wide-field inverted fluorescence microscope, a PLAN APO × 60 oil, 1.42 NA objective and a Photometrics CoolSNAP HQ2 camera at 28 °C. Images were projected and deconvolved using SoftwoRx software. Merges and brightness/contrast modifications were done using the FIJI version of Image J.

### Genomic DNA extraction and quantitative PCR

Genomic DNA was prepared using phenol-chloroform extraction from overnight cultures grown in YE4S at 32 °C. Concentration of the samples was normalized to 50 μg μl^−1^ and 75 μg were used as a template for PCR. qPCR reactions using *cdc13* and *act1* ORF oligos were carried out using EXPRESS SYBR GreenER (Invitrogen) according to the manufacturer's recommendations in an ABI 7500 Fast qPCR machine. Gene copy number was quantified using the standard curve method.

### Cell size measurements

Cell size was measured from images of Blankophor-stained cells (Sigma) using the PointPicker plug-in of ImageJ (National Institutes of Health). For each strain 100 cells were measured under the microscope.

## Author contributions

P.G.-E. designed and carried out the experiments. Both authors discussed the experiments and co-wrote the manuscript.

## Additional information

**How to cite this article:** Gutiérrez-Escribano, P. *et al.* A single cyclin–CDK complex is sufficient for both mitotic and meiotic progression in fission yeast. *Nat. Commun.* 6:6871 doi: 10.1038/ncomms7871 (2015).

## Supplementary Material

Supplementary Information Supplementary Figures 1-5 and Supplementary Tables 1-2
