# Supplementary material for: A single cyclin–CDK complex is sufficient for both mitotic and meiotic progression in fission yeast
Source: Nat Commun. 2015 Apr 20;6:6871. doi: 10.1038/ncomms7871 (PMC4411289; doi:10.1038/ncomms7871)
Supplement: Supplementary Information — Supplementary Figures 1-5 and Supplementary Tables 1-2 [file ncomms7871-s1.pdf]

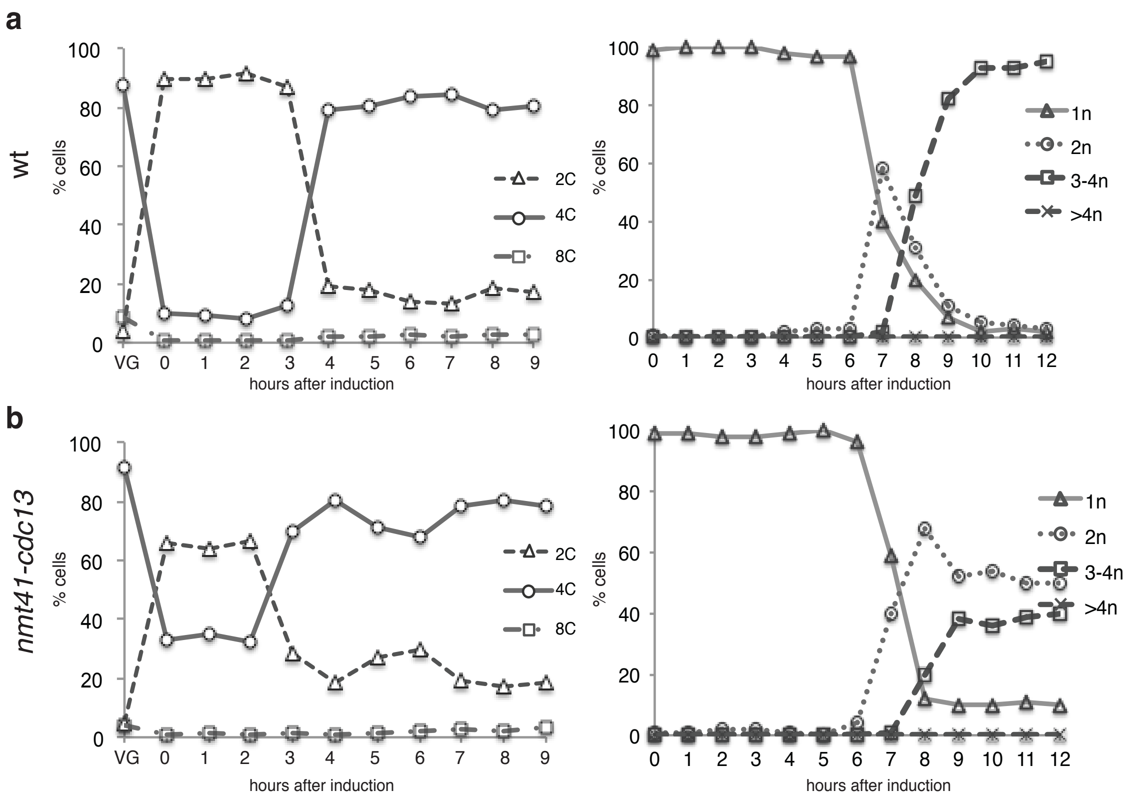

Supplementary Figure 1. Synchronous azygotic meiosis in non-repressive conditions in an *nmt41-cdc13* strain. Synchronous meiosis was chemically induced in **(a)** h+/h+ *pat1as2* (wt) and **(b)** h+/h+ *Δ*cdc13 *nmt41-cdc13 pat1as2 (nmt41-cdc13)* homozygous diploid strains. Pat1 inactivation was achieved by adding 30µM of Nm-PP1 at 28ºC to the culture after 17 hours of nitrogen starvation (time 0). No thiamine was added to the media to allow *cdc13* expression under *nmt41* promoter. DNA content (left panel) and the number of nuclei (right panel) were followed every hour.

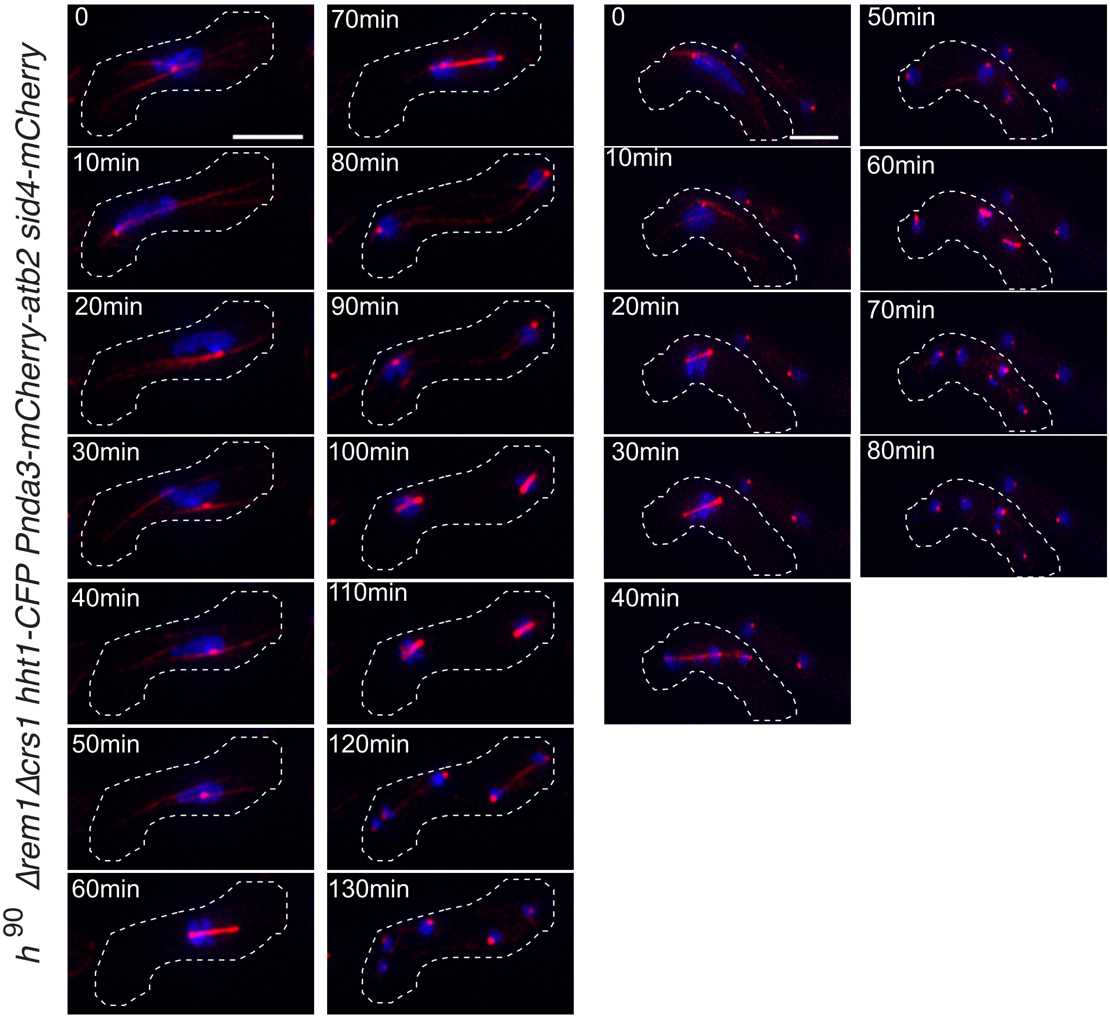

Supplementary Figure 2. *Δrem1Δcrs1* mutants show multipolar divisions during zygotic meiosis. Time-lapse of *h^{90} hhtc1-CFP atb1-mCherry sid4-mCherry rem1Δcrs1Δ* strain (Pi275) after meiotic induction. Images show a representative example of multipolar division during meiosis II (left panels) and meiosis I (right panels). These aberrant divisions were more frequent during meiosis II (45.4%) than during meiosis I (36.4%), while 18.2% of these zygotes showed defects in both meiosis I and II. Scale bars, 5μm.

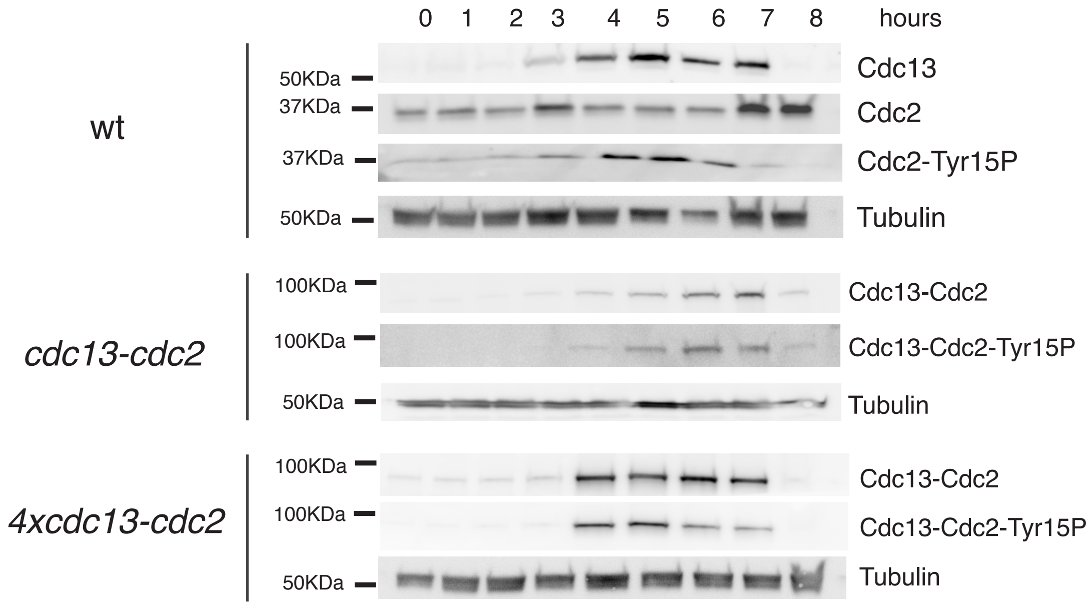

Supplementary Figure 3. Cdc13-Cdc2 fusion protein dynamics during synchronous meiosis. Cells from the experiment described in Figure 7a and 7b were collected every hour to follow protein dynamics. A diploidized wild type strain was used as a control (Pi263). Protein samples were analysed by Western blot using anti-Cdc13 (SP4), Cdc2 (PSTAIRE) and Cdc2-Tyrosine15 phospho-specific antibodies in the case of wt extracts and anti-Cdc13 and Cdc2-Tyrosine15 phospho-specific antibodies for *cdc13-cdc2* samples. α-Tubulin was used in both cases as loading control.

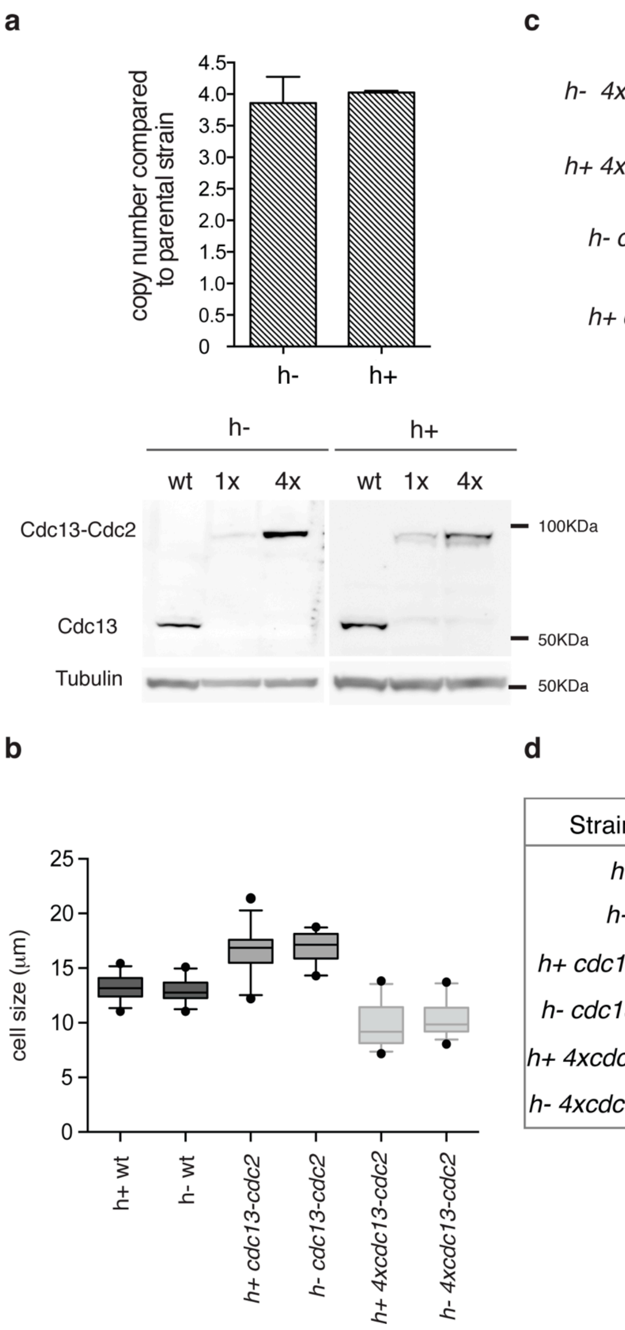

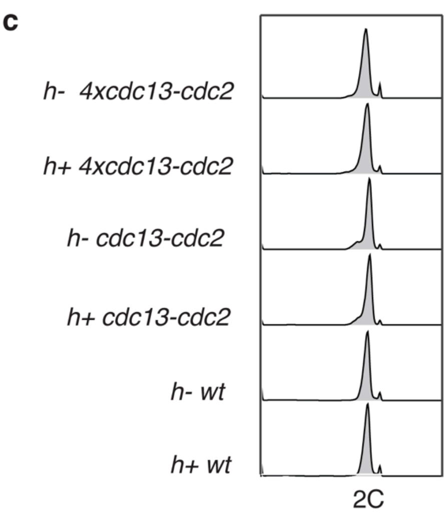

d

Strain	Generation time (min)
h+	125±7
h-	122±3
h+ cdc13-cdc2	133±4
h- cdc13-cdc2	125±7
h+ 4xcdc13-cdc2	134±12
h- 4xcdc13-cdc2	143±9

Supplementary Figure 4. Characterization of the mitotic phenotype of *cdc13-cdc2* over-expression. a. qPCR quantification of *cdc13-cdc2* copy number in *h+* (Pi46) and *h-* (Pi43) *cdc13-cdc2* over-expressing strains, normalized using the qPCR values of *h+* and *h- cdc13-cdc2* parental strains. Protein levels were analysed by Western blot in the same strains using anti-Cdc13 (SP4) and anti-tubulin (TAT1) antibodies, and including *h-* and *h+* wild type strains as controls. **b.** Size at division**,** (n=80 cells per strain, boxes represent 25–

75% of the data and the central lines indicate the median. Whiskers show 5–95% of the data. Dots represent the outter 10%). **c.** FACS profiles. **d.** generation times (mean of three experiments ± standard deviation) of *h+* (PN4) and *h-* (PN1) wt, *cdc13-cdc2 Δ2 Δ13 ΔCCP* (*cdc13-cdc2, h+* DC235 and *h-* Pi4) and 4x *cdc13-cdc2 Δ2 Δ13 ΔCCP* (4x*cdc13-cdc2, h+* Pi46 and *h-* Pi43*)* strains grown at 32ºC in YE4S.

a

	wt	*cdc13-cdc2 Δ2Δ13ΔCCP*	*4xcdc13-cdc2Δ2Δ13* *ΔCCP*	*4xcdc13-cdc2Δ2Δ13* *ΔCCPR*	*4xcdc13-cdc2Δ2Δ13* *ΔCCPRC*
**> 4 DAPI stained bodies**	1.8	1.2	3.5	2.6	3.7
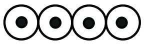	89.3	3.4	90.2	87	85.7
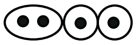	0.9	2.2	-	2.6	0.5
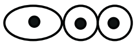	4.5	3.4	3.3	1	1.2
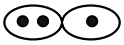	1.3	6.7	-	1.6	1.7
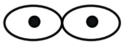	2.2	82	3	4.1	5.6
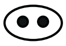	-	-	-	1.1	0.5
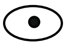	-	1.1	-	-	1.1

b

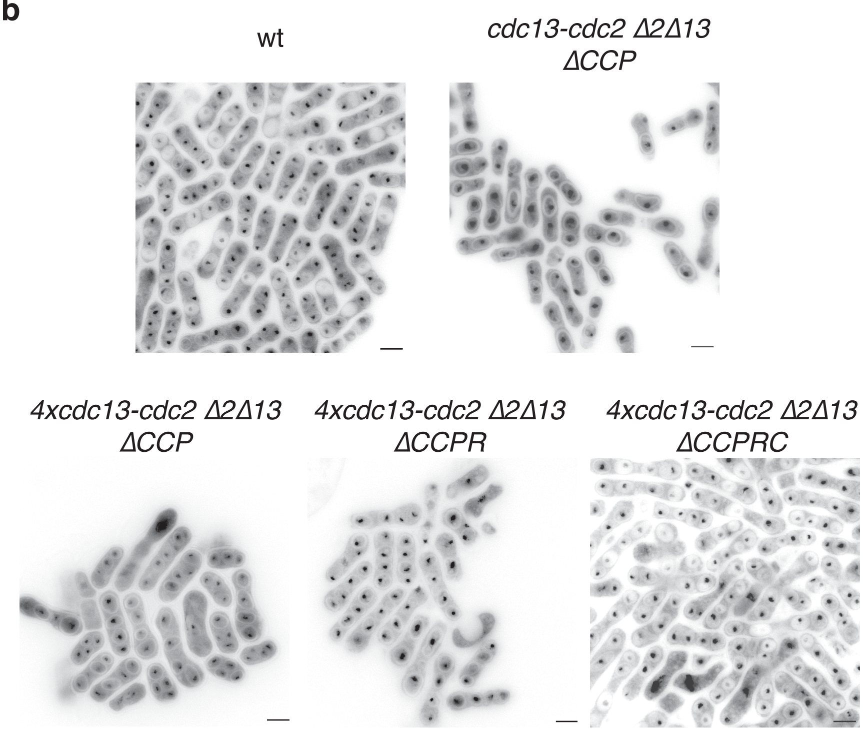

Supplementary Figure 5. Frequency of aberrant nuclear divisions in Cdc13-Cdc2 fusion protein strains during meiosis. Asci from a*zygotic* synchronous meiosis were stained with DAPI and observed by microscopy. **a**. Phenotipic defects considered (based on [20]) and percentage of asci in each of them. At least 400 asci were scored for each strain. b. Representative pictures of each strain after 14 hours of meiotic induction. Scale bars, 5μm.

Supplementary Table 1. Strains used in this study

**Name**	**Genotype**	**Source**
PN1	*h⁻* 972	Lab collection
PN4	h^+ 975	Lab collection
PN71	h^- *ade6-M210 leu1-32*	Lab collection
PN1394	h^- *Δcig1 ::ura4+ Δcig2 ::ura4+ ade6- leu1-32 ura4-D18*	Lab collection
PN1400	h^- *Δcig1 ::ura4+ Δcig2 ::ura4+ ura4-D18*	Lab collection
PN1401	h^+ *Δcig1 ::ura4+ Δcig2 ::ura4+ ura4-D18*	Lab collection
PN1926	h^+ *Δcig2 ::ura4+ ura4-D18*	Lab collection
PN1931	h^- *Δcig2 ::ura4+ leu1-32 ura4-D18*	Lab collection
PN1942	h^- *Δcig2 ::ura4+ ura4-D18*	Lab collection
DC177	h^- *leu1 ::Pcdc13::cdc13-L-cdc2::cdc13 3'UTR::ura4+ Δcdc2 ::kanMX6 Δcdc13::natMX6 ura4-D18*	[4]
DC210	h^+ *leu1 ::Pcdc13::cdc13ts-L-cdc2as::cdc13 3'UTR::ura4+ Δcdc2 ::kanMX6 Δcdc13::natMX6 ura4-D18*	[4]
DC235	h^+ *leu1 ::Pcdc13::cdc13-L-cdc2::cdc13 3'UTR::ura4+ Δcdc2 ::kanMX6 Δcdc13::natMX6 Δcig1 ::ura4+ Δcig2 ::ura4+ Δpuc1 ::ura4+ ura4-D18*	[4]
Pi1	h^- *Δcig1::ura4 Δcig2::ura4 Δpuc1::ura4 ura4-D18*	This work
Pi4	h^- *leu1 ::Pcdc13::cdc13-L-cdc2::cdc13 3'UTR::ura4+ Δcdc2 ::kanMX6 Δcdc13::natMX6 Δcig1 ::ura4+ Δcig2 ::ura4+ Δpuc1 ::ura4+ ura4-D18*	This work
Pi5	h^+ *Δcig1::ura4 Δcig2::ura4 Δpuc1::ura4 ura4-D18*	This work
Pi43	*h- leu1 ::Pcdc13::cdc13-L-cdc2::cdc13 3'UTR::ura4+ Δcdc2 ::kanMX6 Δcdc13::natMX6 Δcig1 ::ura4+ Δcig2 ::ura4+ Δpuc1 ::ura4 3x(ura4+:: Pcdc13::cdc13-L-cdc2::cdc13 3'UTR::ura4+::Scleu2) ura4-D18*	This work
Pi46	h^+ *leu1 ::Pcdc13::cdc13-L-cdc2::cdc13 3'UTR::ura4+ Δcdc2 ::kanMX6 Δcdc13::natMX6 Δcig1 ::ura4+ Δcig2 ::ura4+ Δpuc1 ::ura4 3x(ura4+:: Pcdc13::cdc13-L-cdc2::cdc13 3'UTR::ura4+::Scleu2) ura4-D18*	This work
Pi130	h^- *leu1 ::Pcdc13::cdc13-L-cdc2::cdc13 3'UTR::ura4+ Δcdc2 ::kanMX6 Δcdc13::natMX6 Δcig1 ::ura4+ Δcig2 ::ura4+ Δpuc1 ::ura4 Δrem1::hphNT1 3x(ura4+:: Pcdc13::cdc13-L-cdc2::cdc13 3'UTR::ura4+::Scleu2) ura4-D18*	This work
Pi132	h^+ *leu1 ::Pcdc13::cdc13-L-cdc2::cdc13 3'UTR::ura4+ Δcdc2 ::kanMX6 Δcdc13::natMX6 Δcig1 ::ura4+ Δcig2 ::ura4+ Δpuc1 ::ura4 Δrem1::hphNT1 3x(ura4+:: Pcdc13::cdc13-L-cdc2::cdc13 3'UTR::ura4+::Scleu2) ura4-D18*	This work
Pi155	h^- *leu1 ::Pcdc13::cdc13-L-cdc2::cdc13 3'UTR::ura4+ Δcdc2 ::kanMX6 Δcdc13::natMX6 Δcig1 ::ura4+ Δcig2 ::ura4+ Δpuc1 ::ura4 Δrem1::hphNT1 Δcrs1::hphNT1 3x(ura4+:: Pcdc13::cdc13-L-cdc2::cdc13*	This work

	3'UTR::ura4+::Scleu2) ura4-D18	
Pi158	h^+ leu1 ::Pcdc13::cdc13-L-cdc2::cdc13 3'UTR::ura4+ Δcdc2 ::kanMX6 Δcdc13::natMX6 Δcig1 ::ura4+ Δcig2 ::ura4+ Δpuc1::ura4 Δrem1::hphNT1 Δcrs1::hphNT1 3x(ura4+:: Pcdc13::cdc13-L-cdc2::cdc13 3'UTR::ura4+::Scleu2) ura4-D18	This work
163	h+ Δrem1::hphNT1	This work
166	h- Δcrs1::hphNT1	This work
Pi175	h^- Δrem1::hphNT1 Δcrs1::hphNT1	This work
Pi176	h^+ Δrem1::hphNT1 Δcrs1::hphNT1	This work
Pi211	h^+ Δcig1::ur4 Δcig2::ura4 Δpuc1::ura4 Δrem1::natNT2 Δcrs1::hphNT1 ura4-D18	This work
Pi230	h^+/h^- Δrem1::hphNT1/ Δrem1::hphNT1 Δcrs1::hphNT1/ Δcrs1::hphNT1 cut11-GFP::kanmx6/ cut11-mCherry::natmx6	This work
Pi234	h^{90} hht1-CFP:his+ Pnda3-mCherry-atb2:aur1R Sid4-mCherry::Scleu2 leu1-32 his3-D1 ura4-D18	This work
Pi236	h^+ Δcig1::ura4 Δcig2::ura4 Δpuc1::ura4 Δrem1::natNT2 ura4-D18	This work
Pi237	h- Δcig1::ur4 Δcig2::ura4 Δpuc1::ura4 Δcrs1::hphNT1	This work
Pi240	h^+ Δcig1::ura4 Δcig2::ura4 Δpuc1::ura4 Δrem1::natNT2 Δpat1::pat1as2::hph	This work
Pi242	h^- Δcig1::ur4 Δcig2::ura4 Δpuc1::ura4 Δrem1::natNT2 Δcrs1::hphNT1 ura4-D18	This work
Pi251	h^- Δcig1::ur4 Δcig2::ura4 Δpuc1::ura4 Δrem1::natNT2 ura4-D18	This work
Pi253	h^+ leu1 ::Pcdc13::cdc13-L-cdc2::cdc13 3'UTR::ura4+ Δcdc2 ::kanMX6 Δcdc13 ::natMX6 Δcig1 ::ura4+ Δcig2 ::ura4+ Δpuc1 ::ura4+ Δpat1::pat1as2::hph ura4-D18	This work
Pi259	h^+ Δcig1::ura4 Δcig2::ura4 Δpuc1::ura4 Δpat1::pat1as2::hph ura4-D18	This work
Pi260	h^+ leu1 ::Pcdc13::cdc13-L-cdc2::cdc13 3'UTR::ura4+ Δcdc2 ::kanMX6 Δcdc13 ::natMX6 Δcig1 ::ura4+ Δcig2 ::ura4+ Δpuc1 ::ura4 3x(ura4+:: Pcdc13::cdc13-L-cdc2::cdc13 3'UTR::ura4+::Scleu2) Δpat1::pat1as2::hph ura4-D18	This work
Pi263	h^+Δpat1::pat1as2::hph	This work
Pi275	h^{90} hht1-CFP:his+ Pnda3-mCherry-atb2:aur1R Sid4-mCherry::Scleu2 Δrem1::natNT2 Δcrs1::natNT2 leu1-32 his3-D1 ura4-D18	This work
Pi278	h^+ Δcig1::ur4 Δcig2::ura4 Δpuc1::ura4 Δrem1::natNT2 Δcrs1::hphNT1 Δpat1::pat1as2::hph	This work
Pi279	h^- leu1 ::Pcdc13::cdc13-L-cdc2::cdc13 3'UTR::ura4+ Δcdc2::kanMX6 Δcdc13::natMX6 Δcig ::ura4+ Δcig2::ura4+ Δpuc1::ura4 Δrem1::hphNT1	This work

	3x(ura4+:: Pcdc13::cdc13-L-cdc2::cdc13 3'UTR::ura4+::Scleu2) Δpat1::pat1as2::hph ura4-D18	
Pi283	*h⁺ Δrem1::hphNT1 Δcrs1::hphNT1 Δpat1::pat1as2::hph*	This work
Pi285	*h⁺ leu1::Pcdc13::cdc13-L-cdc2::cdc13 3'UTR::ura4+ Δcdc2 ::kanMX6 Δcdc13::natMX6 Δcig1 ::ura4+ Δcig2 ::ura4+ Δpuc1::ura4 Δrem1::hphNT1 Δcrs1::hphNT1 3x(ura4+::Pcdc13::cdc13-L-cdc2::cdc13 3'UTR::ura4+::Scleu2) Δpat1::pat1as2::hph*	This work
Pi292	*h⁺ Δcig1::ur4 Δcig2::ura4 Δpuc1::ura4 Δcrs1::hphNT1*	This work
Pi296	*h⁺ Δcig1::ur4 Δcig2::ura4 Δpuc1::ura4 Δcrs1::hphNT1 Δpat1::pat1as2::hph*	This work
Pi313	*h⁺ Δcdc13::ura4* int*REP41-cdc13::Scleu2 Δpat1::pat1as2::hph*	This work
Pi327	*h- Δcrs1::hphNT1 ade6M26*	This work
Pi328	*h+ Δcrs1::hphNT1 leu1-32 ade6M210*	This work
Pi333	*h+ ade6M26*	This work
Pi334	*h- Δcig1::ur4 Δcig2::ura4 Δpuc1::ura4 leu1-32*	This work
Pi335	*h+ Δcig1::ur4 Δcig2::ura4 pat1as2::hph*	This work
Pi336	*h+ Δcig2::ura4 pat1as2::hph*	This work
Pi337	*h- Δrem1::hphNT1*	This work
Pi339	*h+ Δcrs1::hphNT1*	This work
Pi340	*h+ Δrem1::hphNT1 ade6M26*	This work
Pi344	*h- Δrem1::hphNT ade6M210 leu1-32*	This work
Pi346	*h- ade6M210 leu1-32 Δcrs1::hphNTΔrem1::hphNT1*	This work
Pi348	*h+ ade6M26 leu1+ Δcrs1::hphNT Δrem1::hphNT1*	This work

Supplementary Table 2. List of primers used for quantitative PCR.

Primmer name	Sequence
fw-*cdc13*	ATCATTGCGGGTATGCTCTC
rv-*cdc13*	CTGAAGGGCACATCACCTC
fw-*act1*	CGTCGCTTTGGACTTTGAGC
rv-*act1*	TACCAGGTCCGCTCTCATCA
